# Ultrasound: The Extension of Our Hands to Improve the Management of Thyroid Patients

**DOI:** 10.3390/cancers13030567

**Published:** 2021-02-02

**Authors:** Pierpaolo Trimboli

**Affiliations:** 1 Clinic for Endocrinology and Diabetology, Lugano Regional Hospital, Ente Ospedaliero Cantonale, 6900 Lugano, Switzerland; pierpaolo.trimboli@eoc.ch; 2 Faculty of Biomedical Sciences, Università della Svizzera Italiana (USI), 6900 Lugano, Switzerland

Ultrasonography (US) was introduced in the thyroid field in the 1980s to guide the biopsy of palpable, scintigraphically cold nodules. Within a few years, US-guided fashion became the only modality to perform an optimal sampling of a thyroid lesion, and a significant decrease of unnecessary surgeries was recorded [1]. Subsequently, with the technological advancements of medical devices, the US examination of both thyroid and neck rapidly diffused and all thyroidologists, endocrinologists first, began to visit their patients with US alongside the physical examination and laboratory tests. US allows us to estimate the thyroid size, evaluate its echostructure and echogenicity, investigate visible and/or palpable thyroid nodules, and detect non-palpable ones. More importantly, the risk of malignancy of any thyroid lesion and the presence of neck lymph nodes metastases from thyroid cancer could be assessed [2]. Then, it seemed clear to all thyroidologists that US, due to its characteristics, had the potential to become the “extension” of their hands much more than the other imaging tools. At the turn of the 2000s, US examination was also integrated by color-flow Doppler analysis, elastosonography, and contrast-enhanced modality (CEUS) with the aim to detect thyroid carcinoma with higher accuracy [3,4,5]. Overall, since then, it came to light that US was essential to achieve an optimal standard of care of thyroid patients [6] and there was a terrific increase of studies reporting excellent reliability of US to diagnose thyroid cancer. Based on this literature, the US presentation of thyroid cancer is now well recognized and the presence of specific US features (i.e., strong hypoechogenicity, taller-than-wide shape, irregular or blurred margins, internal microcalcifications, apparent extrathyroidal extension) represent an important warning requiring biopsy. More recently, several attempts have been made to further improve the performance of US evaluation and some US-based risk stratification systems (RSSs) have been proposed by the most important international societies. These RSSs, often referred to as thyroid imaging reporting and data system (TIRADS), have been developed to establish a standard lexicon to describe the thyroid nodules, assign nodules to a malignancy risk class, and identify nodules requiring biopsy. The evidence-based studies indicate that the performance of RSSs is close to optimal [7,8]. However, some weaknesses might be present with their rigorous use and further improvements are needed. Particularly, the RSSs have been conceived starting from 20-year literature mainly focused on the US presentation of papillary carcinoma [9] and whether they are reliable to identify follicular and medullary thyroid cancers remains to be proven. Moreover, what will be the role of color-flow Doppler, elastosonography, and CEUS in the era of RSSs has to be defined.

Soon, thyroid US RSSs/TIRADSs will be used by all thyroidologists. Endocrinologists, surgeons, radiologists, nuclear medicine physicians, and cytopathologists focused on thyroid disease will have to be familiar with RSSs/TIRADSs terminology, as was the case when the cytological systems were introduced in clinical practice in 2000s. However, before using RSSs/TIRADSs in a multidisciplinary modality, we need further proofs and this special issue will try to address many of the current questions. Highly experienced thyroidologists focused on US are asked to contribute to this honorable aim.
